# Computer Simulations of Injection Process of Elements Used in Electromechanical Devices

**DOI:** 10.3390/ma15072511

**Published:** 2022-03-29

**Authors:** Adam Gnatowski, Agnieszka Kijo-Kleczkowska, Jaroslaw Krzywanski, Przemyslaw Lemanski, Elzbieta Kopciuszewska

**Affiliations:** 1 Faculty of Mechanical Engineering and Computer Science, Czestochowa University of Technology, 42-201 Czestochowa, Poland; adam.gnatowski@pcz.pl (A.G.); a.kijo-kleczkowska@pcz.pl (A.K.-K.); lemanski@ipp.pcz.pl (P.L.); 2 Faculty of Science and Technology, Jan Dlugosz University in Czestochowa, 42-200 Czestochowa, Poland; 3 Faculty of Art, Technology and Communication, Vistula University, 02-787 Warsaw, Poland; e.kopciuszewska@vistula.edu.pl

**Keywords:** computer simulations, injection molding process, process parameters

## Abstract

This paper presents the computer simulations of the injection process of elements used in electromechanical devices and an analysis of the impact of the injection molding process parameters on the quality of moldings. The study of the process was performed in Autodesk Simulation Moldflow Insight 2021. The setting of the injection process of the detail must be based on the material and process technological card data and knowledge of the injection molding machine work. The supervision of production quality in the case of injection moldings is based on the information and requirements received from the customer. The main goal of the analysis is to answer the question: how to properly set up the process of filling the mold cavities in order to meet the quality requirements of the presented molding. In this paper, the simulation was compared with the real process. It is extremely important to optimize the injection, including synchronizing all process parameters. Incorrectly selected values of the parameters may lead to product defects, leading to losses and destruction of raw materials, and unnecessary energy consumption connected with the process.

## 1. Introduction

The injection molding process is one of the most commonly used plastics processing technologies. It is carried out in injection molding machines and with such auxiliary devices as production thermostats, plastic dryers, grinders for grinding plastics, etc. Many papers studied primary molding conditions, from design creation to product manufacturing [1,2,3,4,5,6,7]. The injection process is influenced by various factors: properties of the material, wear of the mold, temperature fluctuations, parameters of the process [8,9,10,11,12]. Designers of processing machines introduce newer and newer solutions to increase the possibilities of regulating the injection process. In the injection molding process, plastics are processed in a plastic-fluid state. The most critical injection process parameters are temperature, pressure, and injection time. The selection of these parameters depends on the shape and size of the molded part, type and properties of the material used, the efficiency of the injection molding machine, and mold construction [13,14,15,16,17,18].

The material in the cylinder of the injection molding machine is heated thanks to heat supplied by the heating system, heat obtained as a result of frictional resistance of the material during rotation of the screw [9,19]. Mold temperature is the surface temperature of the mold cavities, and its range depends on the type of production and the type of material. In practice, it is assumed 60–120 °C in the production of crystalline materials, 10–20 °C in case of mass-products.

Mold temperature rise increases the crystallization of semicrystalline materials. It improves their usability, increases processing shrinkage and reduces uncontrolled secondary shrinkage, reduces the internal stress of the molded part, reduces collapses and cavities, increases the accuracy of the nest mapping, reduces flow resistance and pressure losses, extends the cooling time (approx. 2%/1 °C) [9,10].

**Figure 1:**
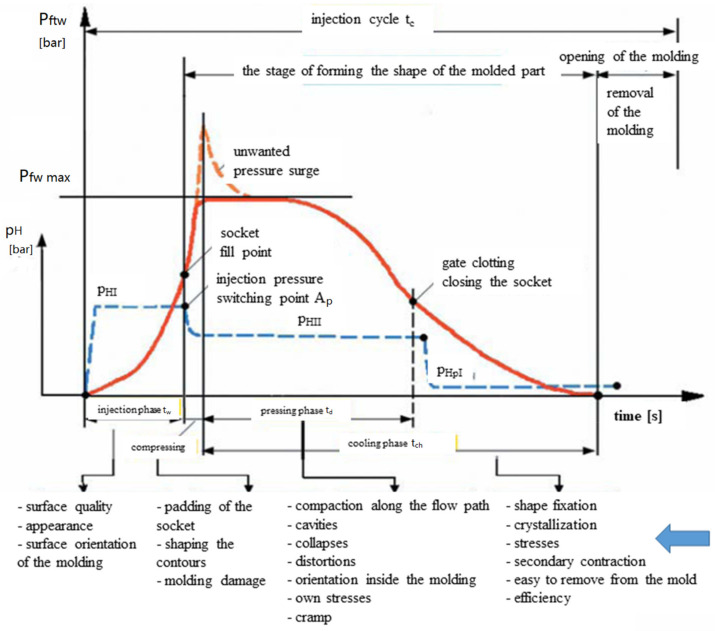
The course of pressure changes in the mold during the injection cycle.

Pressure changes in the injection mold and the accompanying pressure changes in the hydraulic system are shown in Figure 1. In the injection phase, the pressure in the hydraulic system (pHI) reaches its maximum value (approx. 10–14 MPa). There is, as a rule, pressure on the forehead of the snail ten times higher in this phase. This is called injection pressure [9,14,20,21].

In the mold, during rapid flow, the pressure of the material changes depending on the flow resistance and is relatively low. However, immediately after filling the cavity, it directly increases. This growth compresses the material and is necessary for the complete filling of the cavity. On the other hand, too low injection pressure may cause shortcomings or temporary stoppages (supercooling) of the material in individual parts of the seats.

The surge in pressure increase can cause stresses of the molding, dimensional dispersion, reverse movement of the material to the cylinder, energy losses.

The only way to prevent an excessive pressure surge is to switch the pressure in the (pHI) hydraulic system to a lower pressure (pHII) at the time of compression. The switching point is the switching point (Ap).

**Figure 2:**
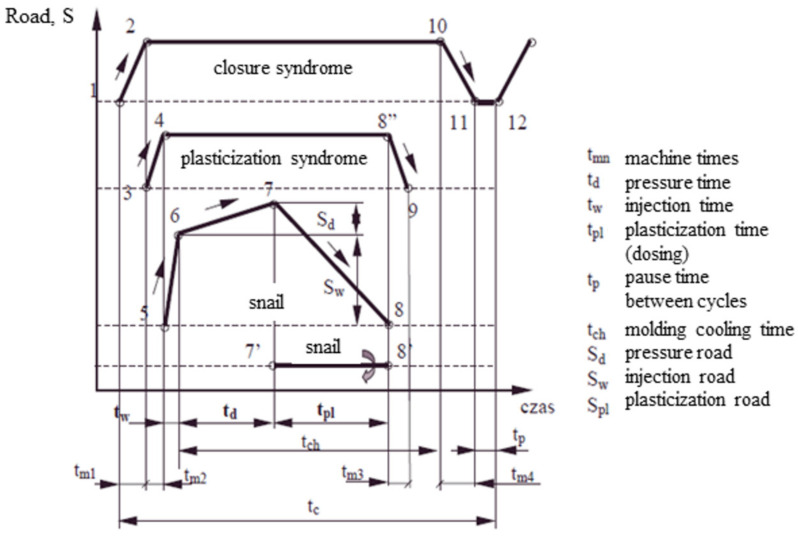
Technological times diagram. Number 1–12 stagest of cycle.

In the injection molding cycle, there are several basic phases resulting from the specific features of the process (Figure 2): injection phase (5–6), pressure phase (6–7), plasticization phase (7–8).

The screw of the injection molding machine, when rotating, automatically moves backward, giving way to the plastic mass moving along its coils. In practice, its free sliding away is difficult because it is counteracted by damping the oil outflow from the working space of the cylinder. In this way, the so-called back pressure is called plasticizing or dispensing pressure. Its value sets the injection pressure at the level of approx. 0.1 [9,16,20].

**Figure 3:**
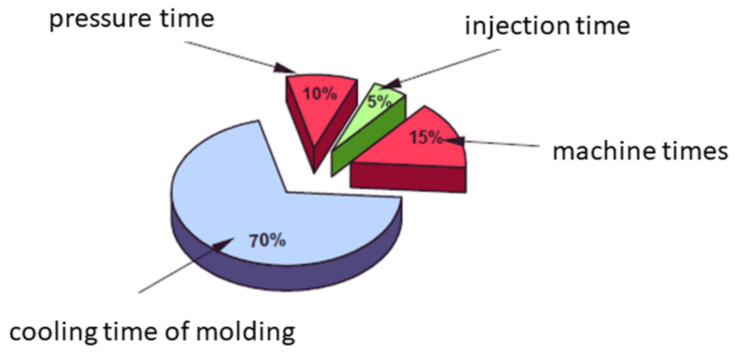
The structure of the most important technological times in the injection molding process.

When analyzing the economic aspects of the production of plastic moldings, it is stated that the essential component of the total production cycle time is the cooling time (Figure 3).

**Figure 4:**
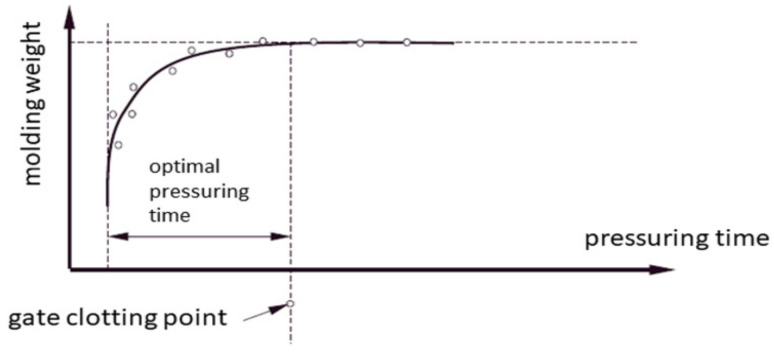
Weight method of determining the pressing time.

Figure 4 presents a graph that determines the minimum time, the exceeding of which does not result in a noticeable increase in the mass of moldings. This time is taken as the optimal pressing time [9,21,22].

**Figure 5:**
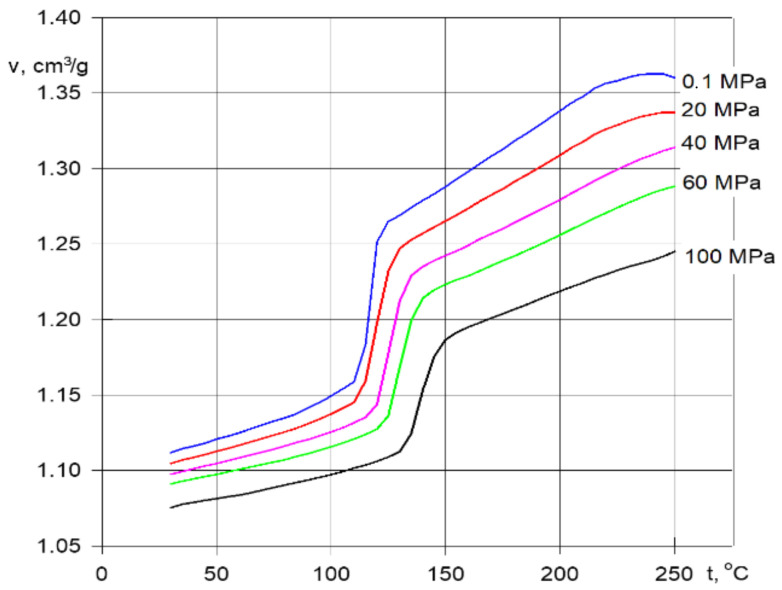
p-v-T plot of polypropylene.

The p-v-T diagram illustrates the changes in the material’s properties during the injection process (Figure 5). The starting point is the variable state of the material in the mold, defined by changes in pressure p, temperature T, and flow velocity v [9,21,23].

It can be concluded that the impact of pressure and the movement of the injection piston may increase the temperature of the material, there is a dependence of the internal pressure in the mold on the external pressure of injection for different temperatures of the material, as the injection temperature increases with constant external pressure, the internal pressure also increases, an increase in the external pressure causes an increase in the sealing pressure, an increase in mold temperature increases the time of the entire cycle.

The specific phenomena may occur in the sequence determined by the course of the cycle and the nature of the behavior of the material, both in the liquid state and during solidification in the mold space, as well as in the solid-state—as secondary shrinkage—for several hundred hours after removing the molded part from the mold [24].

**Figure 6:**
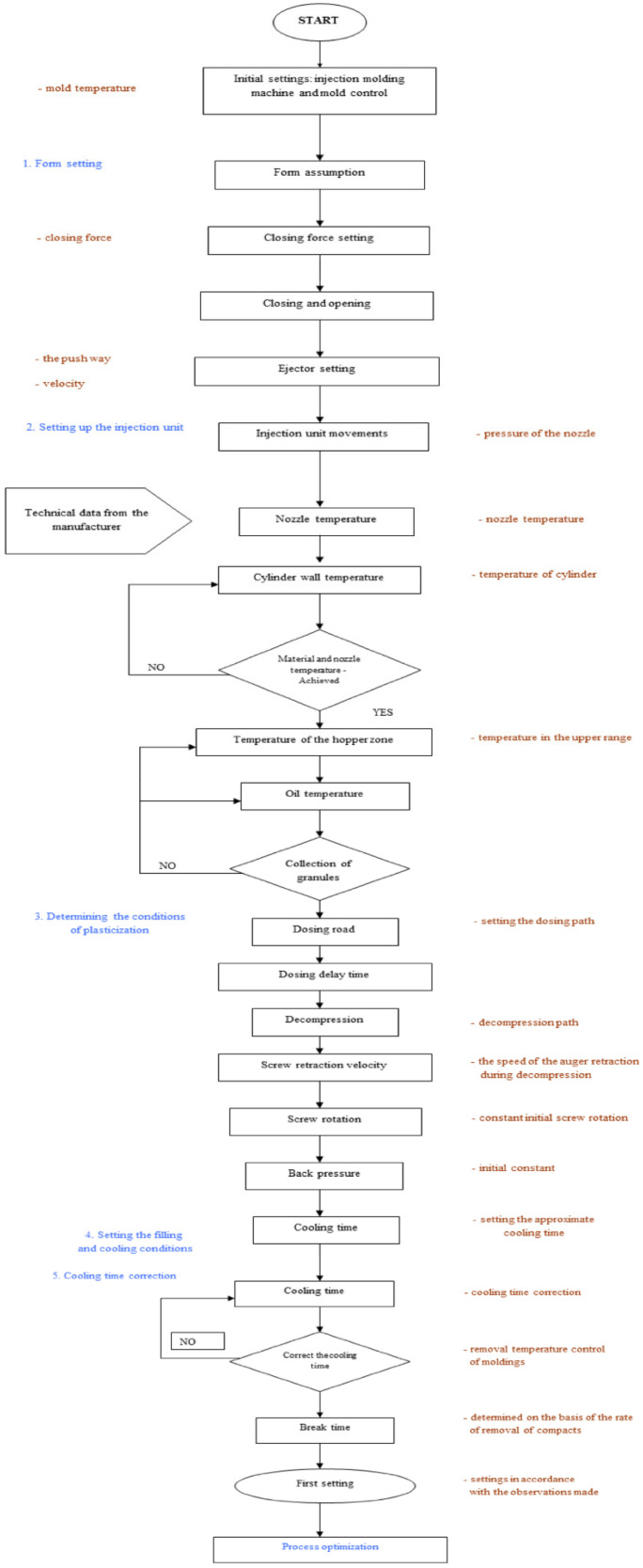
Block diagram of the injection process parameters setting.

Plastic injection molding is a cyclic process. The granular or powder material is plasticized in the cylinder of the injection molding machine by heat and then injected by a screw or piston into the molding cavities. There, the material solidifies at a reduced or increased temperature, maintaining the shape of the finished product—the molded part. After opening the mold, it is removed, and the process can be carried out again. This process is intended mainly for the processing of thermoplastics but is also used for the processing of thermo- and chemo-curable plastics. Injection molding is the basic process of manufacturing finished plastic products weighing from 0.01 g to 70 kg. Figure 6 shows a block diagram of the injection process parameters settings [9,16,20,21].

A number of factors influence the quality of the molded parts: initial preparation of the raw material (drying), its quality (impurities), injection process parameters, the condition of the injection molding machine, level of its wear, arrangement of the hot runner system (cross-section of the channels, places where the material is deposited, etc.), arrangement of supply channels, the possibility of stopping the plugs from the nozzle, venting, etc., the share of pigments and dyes, the type of material being processed, preparation and knowledge of the personnel operating the injection molding machine and supervising the course of the technological process.

Table 1 presents the causes of mold defects and corrective actions aimed at correctly optimizing the injection process parameters [9,10,11,12,25,26,27].

The aim of the paper is to analyze the impact of the injection process parameters on the quality of the production carried out in Autodesk Simulation Moldflow Insight 2021, taking into account the technological data of the material and process as well as the operation of the injection molding machine. The main objective of the analysis is to determine the correct configuration of the process to meet the quality requirements of the presented molding, free from defects, presented in Table 1. The paper compares the simulation with the course of a real process and its technological conditions.

## 2. Organization of the Injection Process and Quality Control of Moldings

The quality assessment of the profile produced by the injection method is based on aesthetic control and visual and laboratory tests. The first visual assessment of the detail is made by the technologist when setting the production parameters of a given molded part. Most of the visible defects that may arise in setting up the process are quite easily noticeable, so any possible aesthetic defects that can be detected are corrected on an ongoing basis at the start of production. The injection molding machine operator can visually detect such defects as overspray, ejector marks, flash burns, material overheating, and the accompanying degradation of the material. These problems can usually be corrected by changing the injection parameters or the mold design.

Common problems that degrade the appearance and reduce the strength of moldings include color, craters, overexposure, traces of overheating, insufficient filling of the mold, joining lines in material [9,10,11,28].

The organization and management of injection molding processes are focused on the needs of a specific customer and their satisfaction with the ordered products. Their goal is to improve the effectiveness and efficiency of processes and the quality of moldings. The quality management system model is in the ISO 9001 standard [29].

The production of moldings can be consulted with the customer in accordance with the appropriate product production plan. When planning the implementation of the product, customer requirements and technical specifications are taken into account. Using the FMEA technique, hazard analysis is performed, trying to prevent errors in processes or defects in products. Acceptance criteria are defined for each phase of the process and can be approved by the customer. Based on information collected from the market, competition analysis, comparison with the best products available on the market, and contracts with suppliers of raw materials and with the customer, the quality requirements for products manufactured by the injection method are established. These requirements are based on technologies and applicable regulations. The requirements relate to the quality characteristics of products and the principles of product delivery and after-sales service. In the product/process development planning phase, the requirements not specified by the customer but necessary to ensure proper production or use of the product are defined. In a justified situation, compliance with the customer’s requirements for defining, documenting, and keeping “special characteristics” under control should be ensured. In the case of changes in the requirements/contract, the Quality Management System deals with the transfer of information to the departments affected by the changes.

Product realization phases, which include product and process design, are carried out through a multidisciplinary approach that, when necessary, ensures the involvement of different departments of the company. The interdisciplinary approach allows for the effective: definition, implementation, and tracking of unique characteristics, FMEA development, development, and review of control plans.

The input data for the design of the process are product design output data, productivity goals, process capability and costs, customer requirements, if any, and experiences from previous studies.

Production process design outputs are expressed in a way that can be compared with process design outputs and include process FMEA, drawings and specifications, process layout, control plan, work instructions, process validation criteria and objectives for quality, reliability, maintainability, analysis of measurement systems, error prevention and rapid measurement methods and response to non-compliance in the production process.

Information (drawings, specifications), appropriate production and measurement equipment, work instructions and controls ensure that the planning conditions for production activities are kept under control.

**Figure 7:**
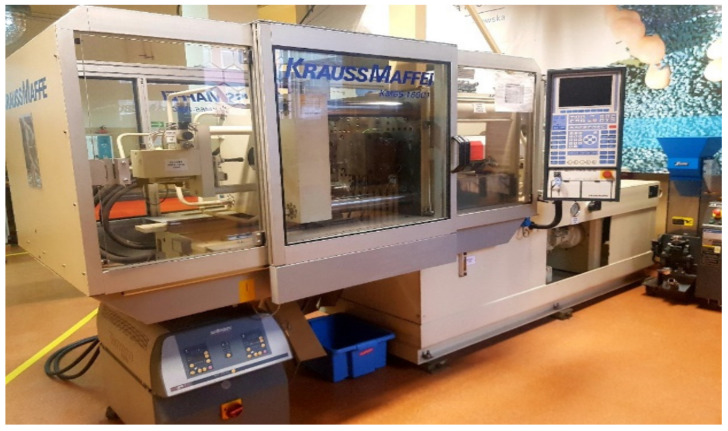
Krauss-Maffei injection molding machine.

The devices that guarantee smooth production and the appropriate quality of the injected moldings include installation of the so-called chilled water (installation for cooling injection molds, injection molding machines and peripheral devices), mold thermo-regulating devices, plastic grinders, dye dosing devices, robots collecting profile from the injection mold. Figure 7 presents the Krauss-Maffei injection molding machine.

The production process requires constant supervision and quality control of products in relation to dimensional tolerance, dimensional stability (SPC), shape (without deformations and sags), workpiece weight tolerance, deformation strength, specific properties in terms of the assumed features of the detailed structure [9,13,14,15,17].

## 3. Analysis of the Injection Process of Detail: A Flowmeter Using the Simulation Analysis of the Injection Process in Moldflow

**Figure 8:**
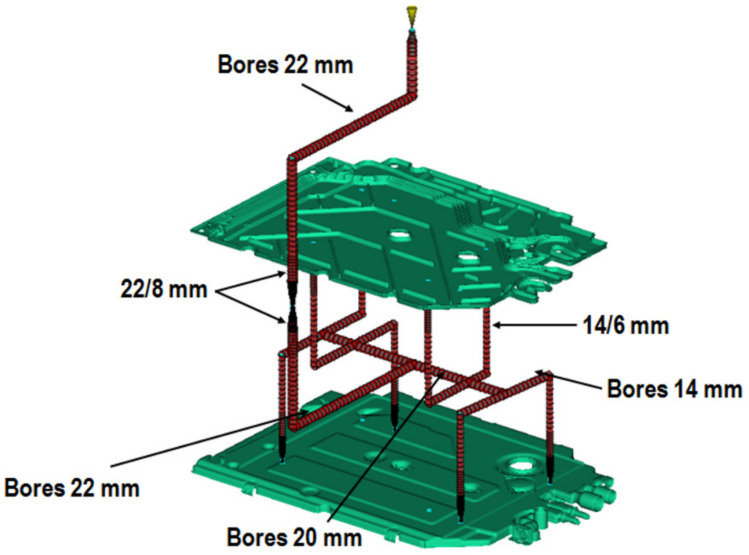
An example of the construction of a plastic feed system in an injection mold.

Having already constructed a tool, an injection mold, and the task of launching serial production of molded parts—the main goal of the analysis is to answer the questions: how to properly set up the process of filling the mold cavities in order to meet the quality requirements of the presented molding (Figure 8). The analysis was performed in Autodesk Simulation Moldflow Insight 2021.

The assumption of the analysis is to obtain a balanced pattern of filling the cavities of the injection mold so as to select the appropriate parameters of the injection process and obtain a satisfactory quality of the molded part. The work includes analysis of the manufactured detail—housing of the flow meter of household appliances. Table 2 presents a summary of the detailed manufacturing parameters.

**Figure 9:**
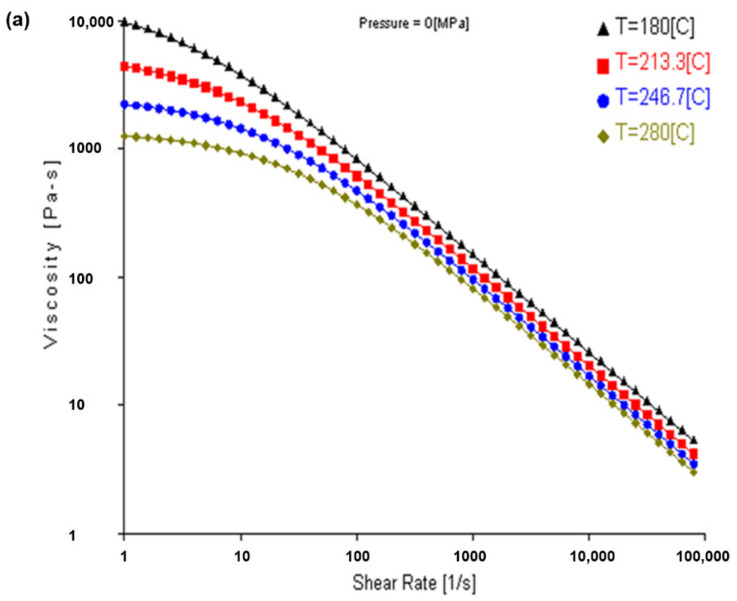

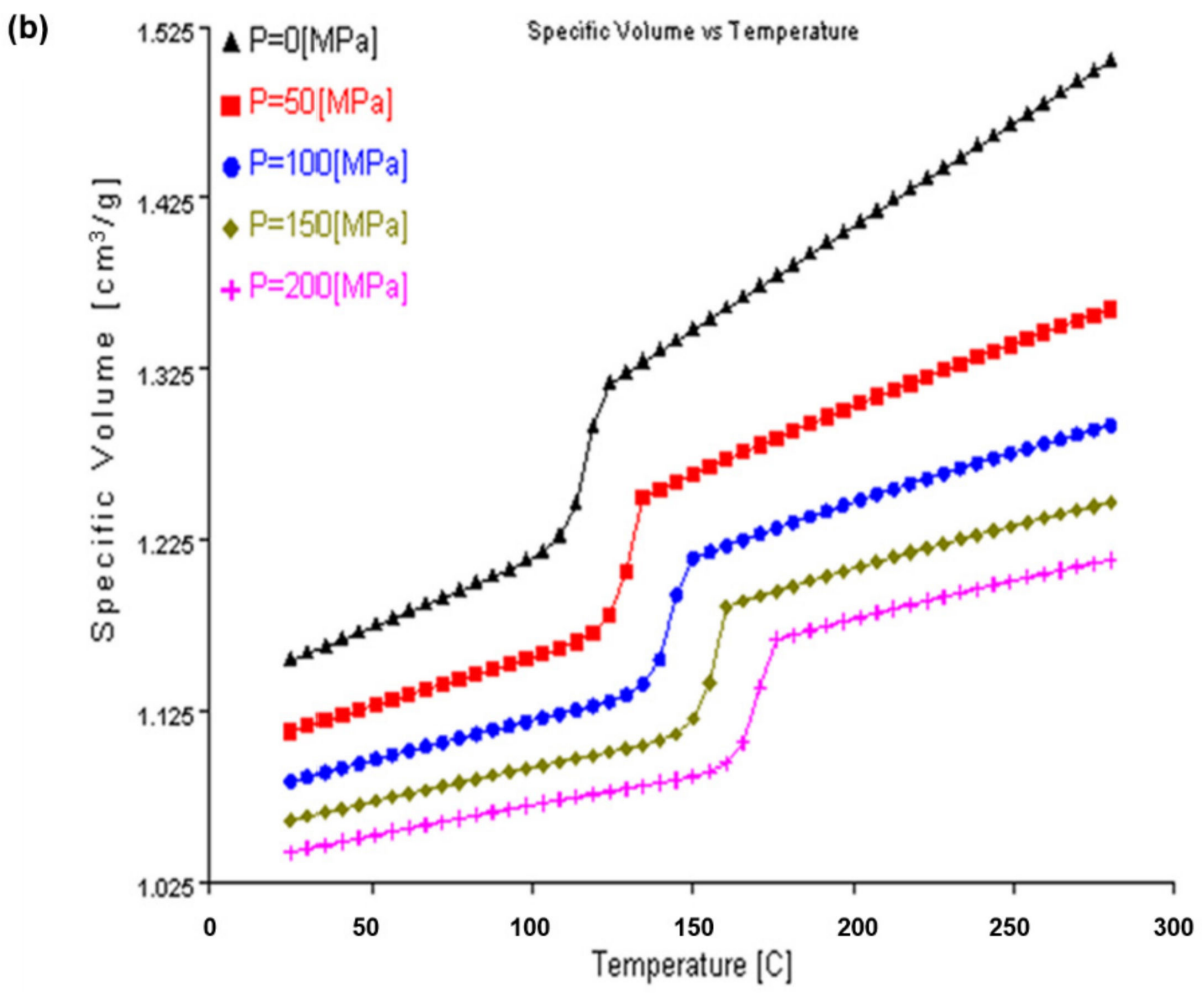
Viscosity—shear rate relationship (**a**) and p-v-T diagrams (**b**).

The p-v-T diagram for the polymer material used is a condensed presentation of the interrelationships of three variables that affect the processing of the polymer: pressure, volume and temperature. The viscosity—shear rate relationship and p-v-T diagrams are illustrated in Figure 9, for amorphous and crystalline polymers.

**Figure 10:**
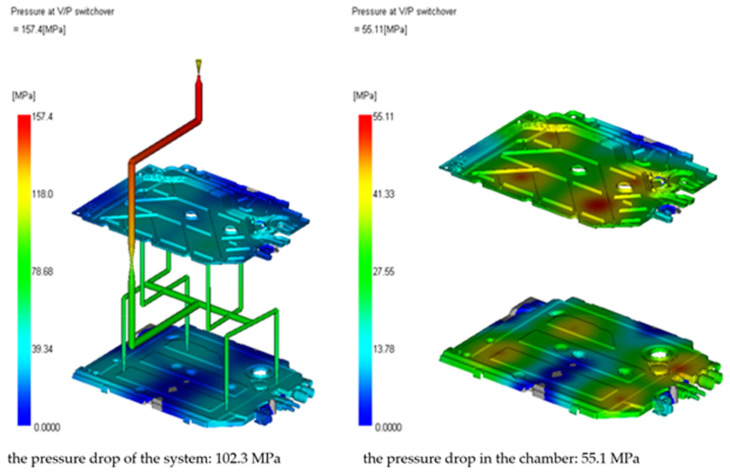
Image of the pressure distribution in the seat at the time of filling of 98%.

In [30], the used material has the technological card. Image of the pressure distribution in the seat at the time of filling of 98% is presented in Figure 10.

**Figure 11:**
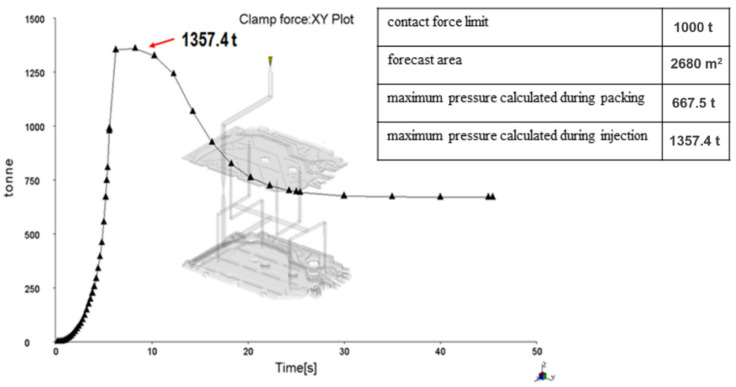
Clamping force calculation.

Figure 11 shows the clamping force calculation. The clamping force is a function of the injection pressure and the protruding seat surface. A well-calculated clamping force should show that the maximum clamping force is lower than the maximum clamping force of the injection molding machine and should not exceed about 75% of the machine’s limit so that the remaining 25% is a safety factor. The requirements of the clamping force usually increase during the pressing of the seat, and therefore, when selecting the injection molding machine, this safety factor should always be taken into account.

**Figure 12:**
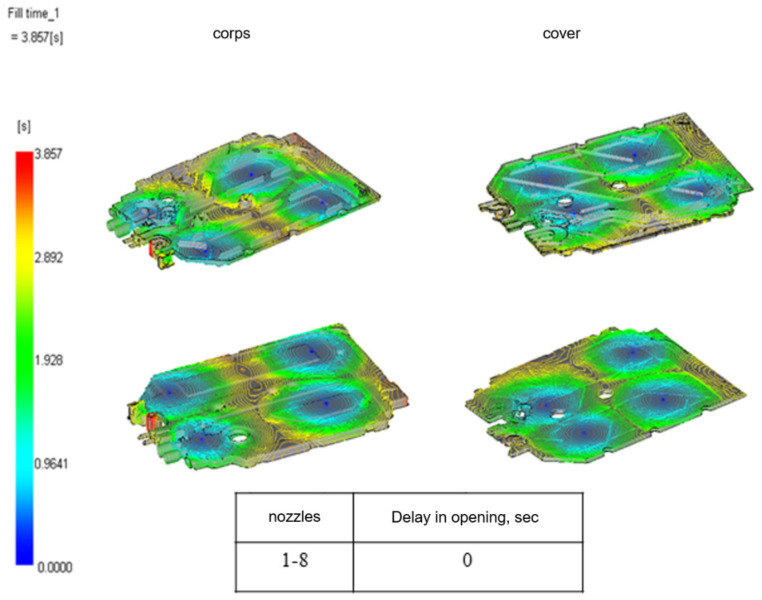
Plastic flow diagrams in the form—visible flow fronts.

Figure 12 shows visible flow fronts. This type of surface makes it easier to identify the swinging areas and highlights the areas where the streams meet. This analysis helps assess, for example, the correctness of the location of points initiating injection into the area of the mold cavity. The image helps to determine whether the distance of initiation points is not too far apart, which is certainly of great importance in setting the injection process and affects not only the aesthetics of the molded part but also results in the variability of the functional properties of the part and the quality of the manufactured product.

**Figure 13:**
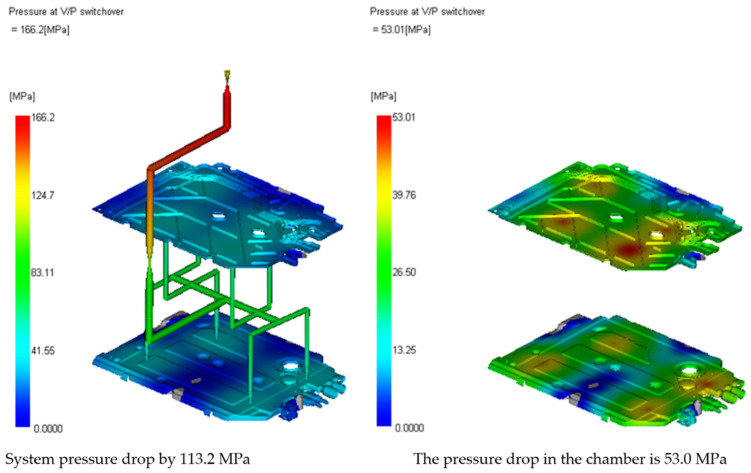
Image of pressure distribution in the seat at the time of filling of 98%.

**Figure 14:**
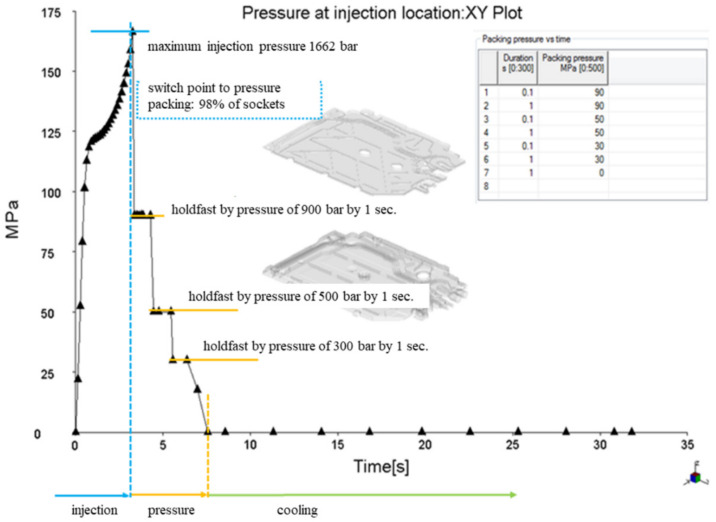
Injection pressure course over time.

**Figure 15:**
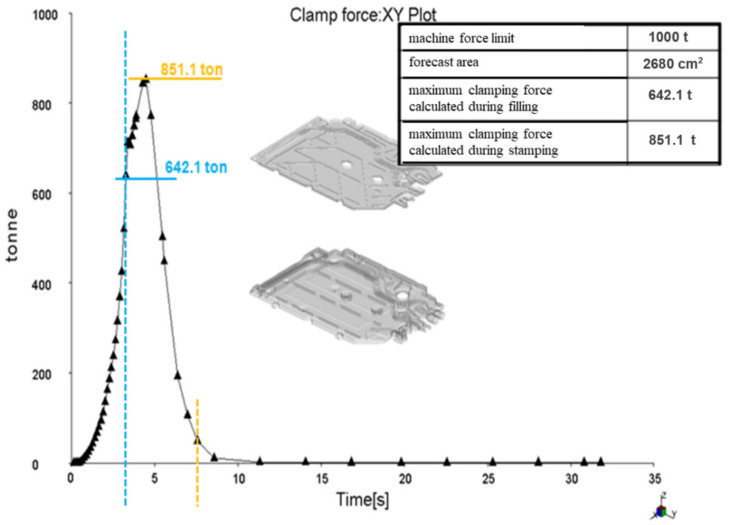
Clamping force calculation.

Figure 13, Figure 14 and Figure 15 present the image of pressure distribution in the seat at the time of filling of 98%, the injection pressure course over time, and the clamping force calculation.

**Figure 16:**
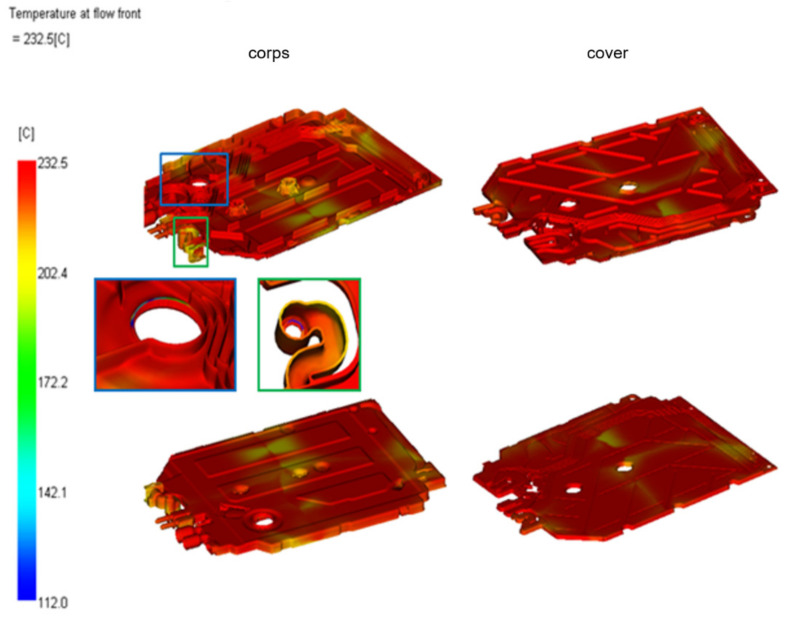
The temperature of the plastic flow front through the socket.

Figure 16 shows the temperature of the polymer when the flow front reaches a certain point (made at the center of the cross-section). The visible difference between the front temperature and the supply temperature by approx. 30 °C.

**Figure 17:**
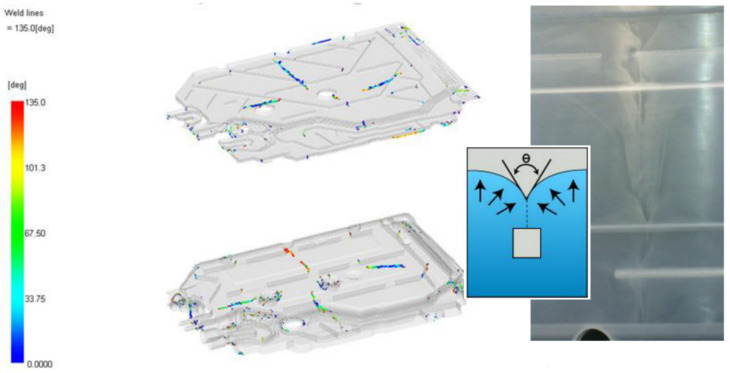
Temperature connection points of streams.

Another analysis performed using Moldflow shows the so-called weld lines—areas (lines) of joining the plastic stream that forms at the meeting angle (θ) from 135 degrees or less. Such a line is very disadvantageous for moldings, not only because of the visual characteristics of the molded part but also because of the weakening of the bonding joint. Figure 17 shows the areas where the streams meet. The color of the welding lines indicates the temperature at which they are formed. Colder temperatures may indicate areas of the difficult seam, which in turn will affect the appearance of the weld line. The strength of the colder seam lines may also be lower than at locations with higher seam line temperatures. The length of the entire weld connecting the lines should be kept to a minimum. Where possible, the injection path should be designed to prevent such areas from forming (Figure 17).

**Figure 18:**
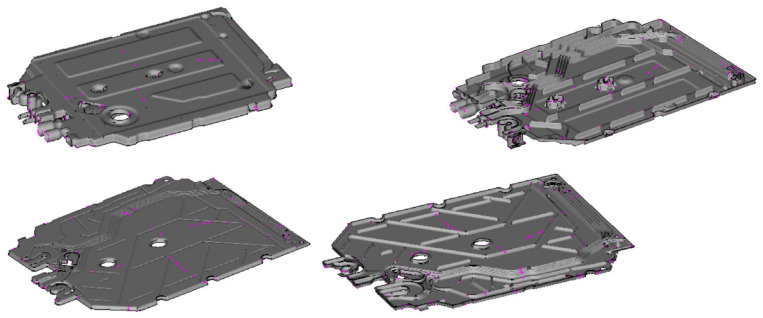
The so-called siphons.

Marked areas indicate the possibility of creating the so-called siphon location (Figure 18). The air which is localized in this place should be eliminated.

Shear rate measures how quickly layers of plastic slide past each other. If this happens too quickly, the polymer chains can break, and the material degrades. The higher shear rate should not exceed the maximum value recommended by the material supplier, and exceeding this value is likely to lead to polymer degradation. It is a good rule not to exceed 60% of the value recommended by the supplier for aesthetic applications.

**Figure 19:**
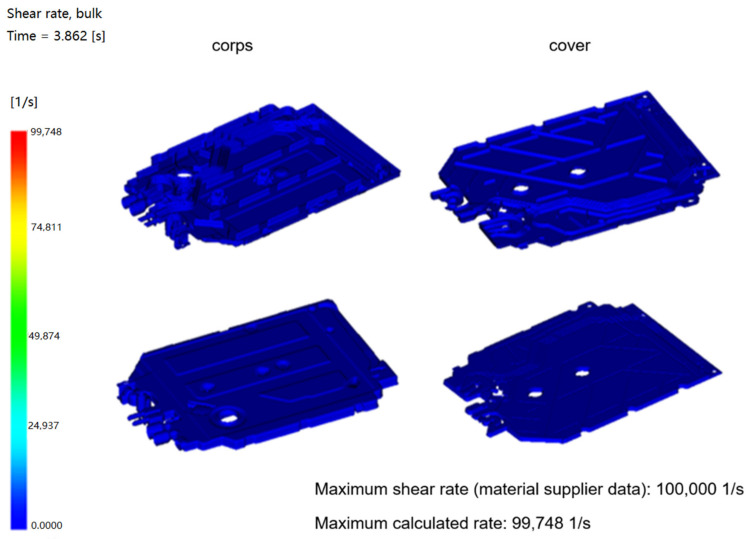
Shear rate.

In the analysis below (Figure 19), you can see a slight difference in the shear rate.

**Figure 20:**
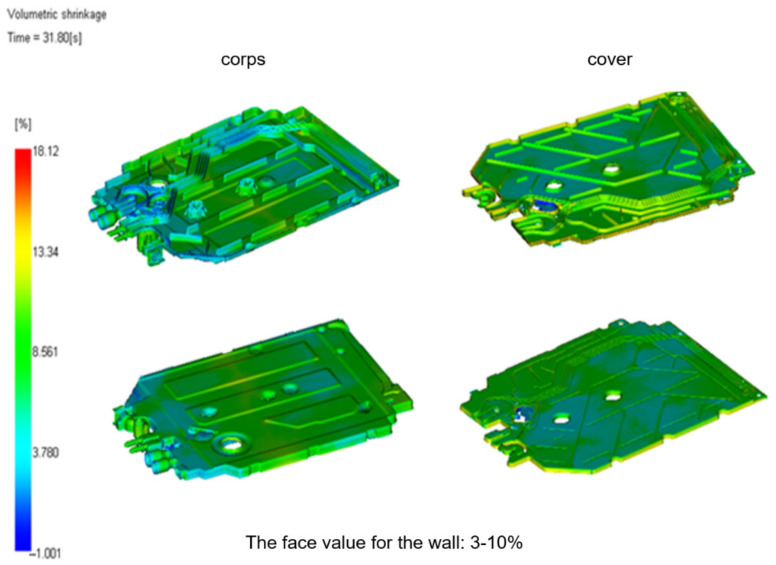
Volume shrinkage of tested moldings of the flow meter.

The behavior of thermoplastics when applying or removing heat is completely different from the known and well-established behavior of other materials. The processing shrinkage of polymers is divided according to several criteria. Due to the time and place of its formation, processing contraction is divided into primary and secondary contraction. Primary shrinkage is understood as reducing the product’s dimensions during its cooling (for thermoplastics) or curing (for hardenable plastics) in the forming cavity of the processing tool and shortly after leaving it. The assumed time of ending the primary contraction is 16 h (Figure 20).

Shrinkage values should be uniform throughout the profile. For example, for a tool that has 0.8% shrinkage, the volumetric shrinkage is 0.8% × 3 = 2.4%. This is essential for good material packing, ensuring good structural and visual integrity of the parts. If the value is greater than three times, it can lead to undesirable deformation of the part and should be assessed in more detail in the warpage analysis.

The above analysis assumed the processing conditions (temperature, pressure with the switching point to the pressure). Considering only the data on the diameter of the plasticizing system nozzle, it is visible how the injection profile of the selected compact changes. The very distinct and noticeable change in flow rate affects the entire shape and illustrates the nest filling processes. It is also impossible not to notice that the very design of the mold, the construction raises minor reservations—the location of the nozzle opening points in the injection mold seat provides many difficulties in setting the correct parameters for the injection process of such a complex molding.

The maximum relative errors between simulation and experimental data are as follows: clamping force 5%, plastic flow in the mold 3%, pressure distribution in the cavity during filling, injection pressure 3%, Pressure drop in the mold 3%, shrinkage 1.5%.

Difficulties in setting and stabilizing the mold injection process, where two moldings have a different shape, are added because the mold is 3-part, and the design of the flow system through the mold can cause considerable difficulties for people who are not experienced in such designs. Therefore, when analyzing the collected materials, it is necessary to thoroughly review the collected data, which will significantly facilitate the assessment of the situation before starting to set the parameters of the injection process of the above-mentioned part.

When setting up the detailed injection process, you also need to consider the order of the material feed channels opening. The practice also shows that not everything can be predicted in setting the parameters on the machine, but very often, they differ from the adopted data for analysis.

**Figure 21:**
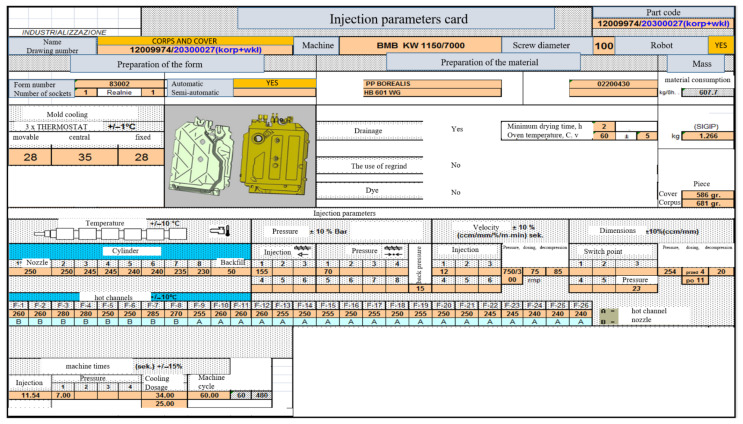
Injection parameters card—moldings of the body and cover of the flowmeter.

Setting the process of injection of the detail must be based on the data provided in the form of material, technological card, knowledge of the injection molding machine, the so-called setting parameters of devices associated with the process (thermostats, heated channel regulators, etc.). Setting up the process also requires familiarization with the construction of the mold, the construction of the cooling system, and hot channels. When initiating a trial batch, the parameters are saved in the so-called memory injection parameter cards (Figure 21).

Supervision of production quality, in this case, injection moldings, is based on the information and requirements received from the customer. Information (drawings, specifications), requirements for instrumentation, production and measurement conditions, work instructions and controls, ensuring the correct course of all production activities play an essential role. For each product a production control plan is developed. The control plan includes output from the product and process FMEA, control of unique characteristics, and response plans when the process becomes unstable. The injection molding product control plan is a living document, i.e., it is updated in the event of changes in the process, product, and in the event of major changes affecting the process capability. Table 3 shows the activities and scope compliant with the control plan.

An important task of the control plan is to fully and clearly express all activities aimed at appropriate verification and control of profile to obtain and maintain customer satisfaction in the end. A product that is new, for example, must have full dimensional documentation of the detail-molded part so that it is possible to determine and make a decision on whether to accept it for serial production. Meanwhile, at each subsequent launch of the production series (according to the order), the dimensional control plan covers only important and essential dimensions, the non-compliance of which may indicate errors in the injection process or, e.g., damage to the injection mold tool, which results in the cessation of production. The restart may take place after eliminating the cause of the incompatibility.

Table 4 and Table 5 and Figure 22, Figure 23, Figure 24, Figure 25, Figure 26, Figure 27, Figure 28 and Figure 29 show the self-check instruction and process.

Another important document for the correct assessment of the injection-molded parts is the card called the “Self-Test Manual”. It is a working instruction intended for operators responsible for producing a given detail. These instructions are closely related to the control plans for a specifically defined molding with other documents of the production process used to assess the manufactured production correctly. Instructions are available at the workstation.

The “Self-inspection” manual contains guidelines for the injection molding machine operator, whose duty is to inspect the workpiece visually. The essence of the instructions mentioned above is:

1. determination of important areas of visual inspection (i.e., marking areas where the non-compliance has occurred or could occur in the future, e.g., the aesthetically unacceptable line connecting the streams of material, tears caused by improper setting of the process of releasing the detail from the mold cavity—traces of ejectors in difficult points, location of the area formulated by hydraulic cores, location of gas traps, etc.),
2. determination of the amount of inspection frequency, which is important, for example, when injection molding parts with low grammage and usually multiple cavities, and a fairly fast cycle. Then the detail does not require 100% inspection. Of course, when inspecting a detail during the process, we analyze a set of working sockets
3. determination of the method and means of control (e.g., only visual inspection or the necessity to use some kind of test).

**Figure 24:**
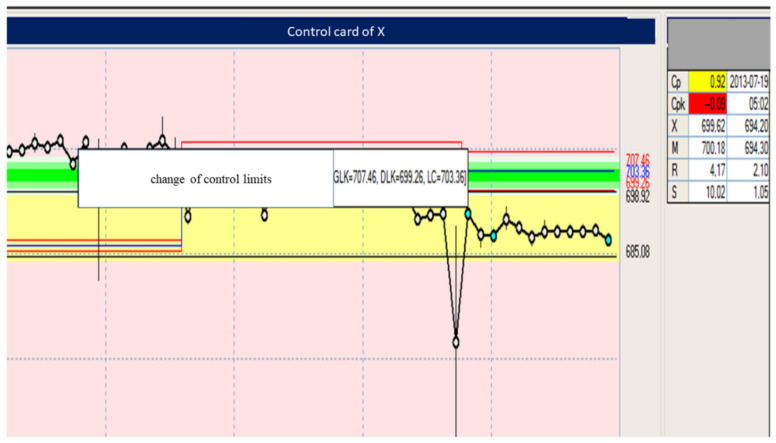
The image showing the parameters adjustment—the effect of reducing the weight of the part.

**Figure 25:**
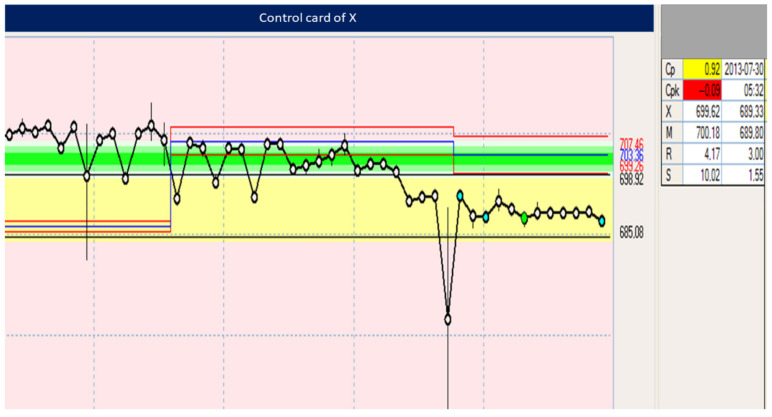
Image showing the onset of injection instability.

**Figure 26:**
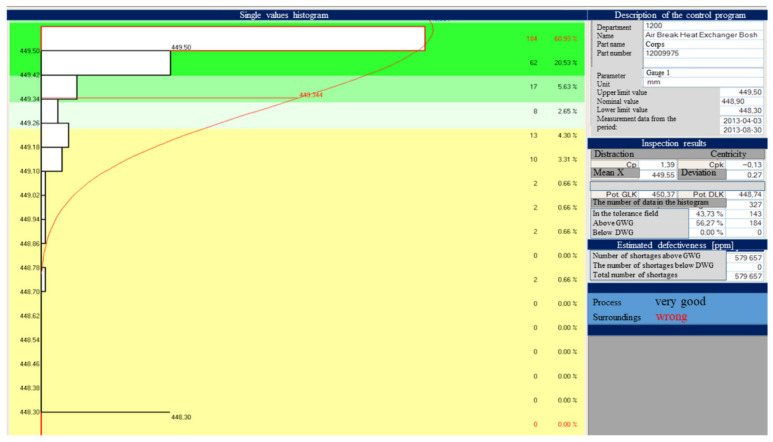
Histogram showing an anomaly in the process.

**Figure 27:**
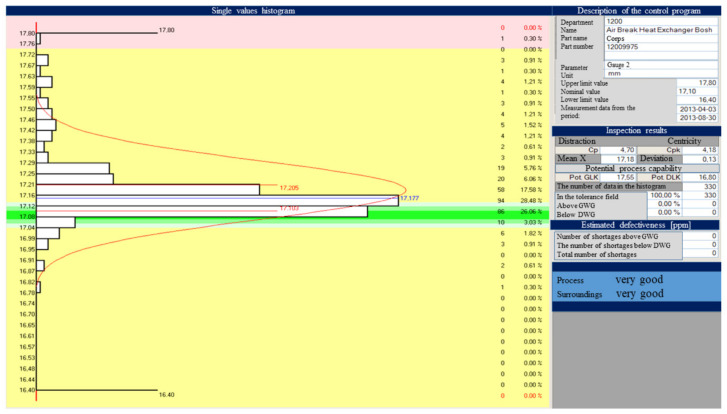
Histogram showing the consistent process.

The above graphs show the beginnings of the process instability. You can also see fluctuations until the parameters and conditions of processing are stabilized. From the moment of changing the parameters, the process has been stabilized, which is shown in the image of the above graph and the histogram of individual values in Figure 24, Figure 25, Figure 26 and Figure 27.

## 4. Conclusions

The molding production process requires the knowledge, precision, and strong commitment of a team of people who, using appropriate devices, can ensure the quality of the required product. It is extremely important to optimize the injection, including synchronizing all process parameters. Incorrectly selecting their values may lead to product defects, leading to losses and destruction of raw materials. Behind the production, success requires good company organization, employee competencies and control, and product verification. The study assessed the injection process on the example of producing a specific detail. The research part presents, on a specific example, the injection process, its course, and, consequently, its impact on the quality of the presented production. A study based on simulations of the injection process was shown, and the simulation was compared with the real process.

## Figures and Tables

**Figure 22:**
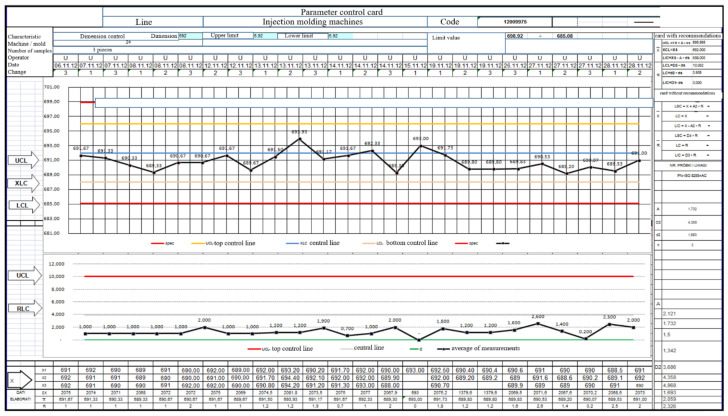
XR card—illustrating the production process of the flow meter detail.

**Figure 23:**
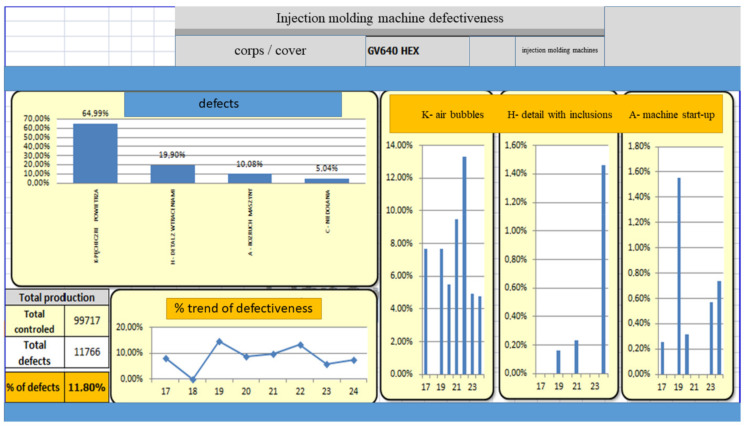
Defect analysis image.

**Figure 28:**
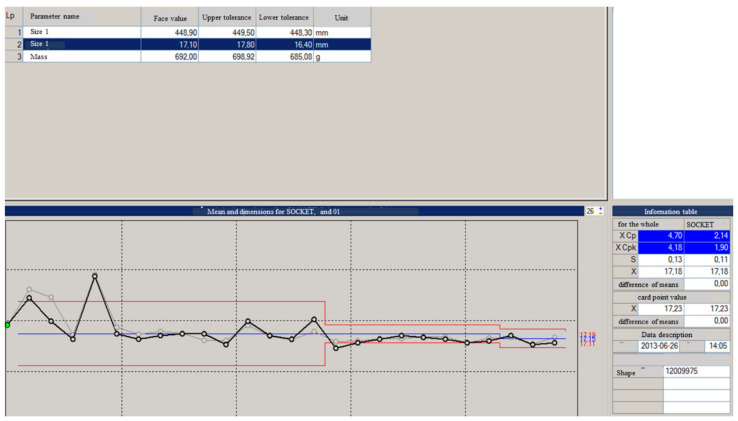
Histogram showing an anomaly in the process.

**Figure 29:**
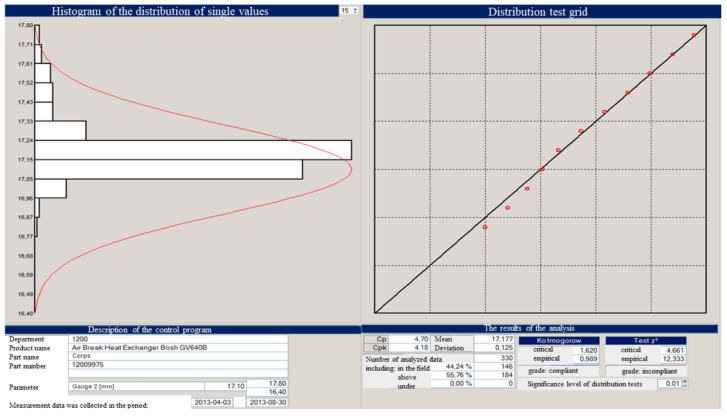
Distribution histogram for single values.

**Table 1:** Reasons for defects in moldings and corrective actions to optimize the injection process.

**Swirls (Jetting)**		
drawback	cause	corrective action
snaky, often rough, or dull streaks appear on the surface of the molded part	this disadvantage occurs when, due to the too high injection speed, while passing through the large cross-sectional area, there is insufficient contact with the wall of the seat, which is necessary for flow laminarity. This defect may result in a deteriorated strength of the shaped body. General causes: material too cold, too cold form, injection speed is too high, which can cause the material or mold to cool too quickly, injection pressures are too high, injection point wrong positioned, the presence of moisture in the plastic	reduce injection speed, increase mold temperaturę, increase the temperature of the hot runner, check the position of the injection point. If necessary, break up the jet with a transverse insert, increase the gate diameter
**Moisture streaks**		
drawback	cause	corrective action
clear, usually matte, elongated, parabolic, white or silvery, emerging on the surface moldings streaks. They are always aimed at the flowing side material	moisture in the plastic, artificial moisture in the mold; the streaks resulting from the moisture contained in the processed material are distinct, usually, matt, elongated, parabolic streaks appearing on the surface of the molded part. Their tips always face the melt flow. The reason for their appearance is the excessive amount of moisture left in the granules of the injected material (insufficiently dry granules) or the water released in the seat or getting into it through leaks in the mold cooling system. The reason for these defects is the presence of fine water vapor bubbles in the plasticizing or injection phase. these bubbles tear at the surface of the mold, and the alloy face flowing at high shear rate creates their elongated and parabolic shape	increase the injection speed, increase the mold temperature, but not above the maximum value recommended by the plastic manufacturer, increase the injection temperature, improve melt homogeneity by increasing the plasticizing pressure (back pressure) and increasing the rotation speed of the screw during dispensing, check the tightness of the mold cooling system, check the accuracy of the packaging of the delivered granulate and its tightness to weather conditions, determine the moisture content in the granules, check the parameters of granulate drying and confront them with those recommended by the material supplier, check the conditions for drying the granulate in the injection molding machine’s hopper. If necessary, reduce the amount of granules in the hopper before the material is taken up by the screw (this means shortening the residence time of the granules in the hopper of the injection molding machine), check the storage conditions of the granulate
**Burns**		
drawback	cause	corrective action
brown or silver streaks on the surface of the part from degraded plastic appear as discoloration	plasticizing temperature too high, the material remains in the plasticizing cylinder for too long, excessive auger speed, abort the cycle without cooling down the cylinder and hot runner, no vents, clogged or misplaced;the reason for this disadvantage is that it is difficult to push the air out of the mold cavity and compress it through the face of the flowing melt. if the air cannot leave the seat through the dividing plane, the clearances between the ejectors and the seat or special venting slots, during this compression it heats up so much that it leads to “burning” of the material surface	check the air ducts for patency, reduce the closing force of the injection molding machine, reduce the injection speed, lower the injection temperature, introduce additional venting channels, change the injection point to ensure that air is released from the socket through the existing venting channels
**Fallsing**		
drawback	cause	corrective action
in case of uneven thickness in its place greater thickness there is a local increase effect contraction volumetric. Layer the surface is pulled inside	differences in thicknessyou of the press, pressure too low Injection, excessive temperature	improve the relationship between areas of different thicknesses on the workpiece, if the defect persists, move the point injection to the largest place thickness, if the collapses are far from the injection point, should be increased pressure and holding time, if the defect persists, the temperature of the plastic and mold must also be increased, if the collapse is close to the injection point, lower the injection and mold temperature as well as the injection speed
**Traces of Stream Joining Lines**		
drawback	cause	corrective action
in points where when filling the nests meet two (or more) melt streams appear similar to scratches or score lines and/or differing in color be gloss areas	the possible causes incomplete fusion: temperature too low, insufficient air or gas evacuation	check the components venting, increase injection speed, increase mold temperature and material, increase clamping pressure
**Colored Streaks**		
drawback	cause	corrective action
they look like shadows in different colors. There are especially visible on materials from metalized or fluorescent pigments	heterogeneous distribution of the material, uneven gates, high auger speed, the temperature of the material is too low, incorrect material mix	check if the applied masterbatch is properly selected for a given material, check the accuracy of the dispenser feeding the color concentrate, compare the concentration of the dye in the concentrate and the material with the values recommended by the masterbatch manufacturer, increase the injection speed, check whether it is possible to shift the injection point or change the wall thickness of the part, check that the melt temperature at the inlet to the sockets is not too high or too low, check, if necessary, that the hot runner temperature is within the range recommended for the processing of a given masterbatch, check the correctness of temperature sensors indications and operation of hot runner temperature regulators, check that the weight of the injection stroke, the number of strokes per minute, and the cylinder volume are in the correct proportion (to do this, compare the time of the material remaining in the injection molding machine with the time recommended by the manufacturer of the material), check if the geometry of the screw is appropriate (its length [L/D], and the recommended grinding, mixing zone, etc.), reduce and increase the rotational speed of the screw during plasticizing, increase the damming (plasticizing) pressure
**An Uneven Gloss of the Part**		
drawback	cause	corrective action
the gloss on the molded part	they can be caused by: non-uniform cooling, deformation of the cooled material, bad form finish	increase clamping pressure, increase holding time, check and, if necessary, polish the mold, raise mold and material temperature, increase injection speed, check the vents for the profile with an invoiced surface: - reduce the injection speed, - reduce the speed of the auger
**Not Added**		
drawback	cause	corrective action
the profile is not completely filled in some places	if unfilled part of the molded part is far from the injection point; the cause may be: insufficient injection volume, insufficient air or gas evacuation, insufficient injection pressure, low injection speed, mold or material temperature too low, valve failure feedback	check and possibly increase the amount of injection material, check the operation of the volatile valve, increase injection speed, increase injection pressure, increase the pressure point, increase mold temperature, increase injection temperature, check the dimensions of the channels and injection points
**Flowed Out**		
drawback	cause	corrective action
they appear when the material squeezes into the gaps in between in halves surface closing the mold, cores	fitting the form elements insufficient to withstand the pressure injection. Possible reasons: form tolerances too large, insufficient strengthclosing the mold, too little stiffness of the boards forming, deformed mold plates, excessive speed injection	increase the mold closing force, or use an injection molding machine of greater clamping force, check for possible deformation, check and, if necessary, change production parameters, reduce injection speed, lower injection and mold temperature, lower injection pressure
**Gas or Air Bubbles**		
drawback	cause	corrective action
profile has blisters inside either on the surface	trapped air orgas, weak internal pressurein the form of badly positioned point injection, gate too small, collapses, screw speed too highinjection molding machines	increase injection pressure. check the diameter of the gateways, reduce injection temperature, check and possibly, reduce the speed of the auger, check for moisture inmaterial granules, check and, if necessary, change the position of the injection point, check whether there are collapses due to shrinkage of the material, check venting
**Black Inclusions**		
drawback	cause	corrective action
dark inclusions in the form of a point or layered	too high injection temperature resulting in material degradation, contaminated material, discarded plasticizing unit, excessive auger speed, excessive back pressure, the presence of impurities in the plasticizing system	clean the machine before changing the material, check whether the powdered pigment or the so-called masterbatch is suitable for the processed polymer, check the tightness of the plasticizing system, contamination from plastic deposits or wear of the plasticizing system, systematically check all—one by one, process elements from opening the container with the plastic through the entire injection molding machine, try to determine at which stage the contamination appears, shorten the storage time of the material in environmental conditions, lower the melt (injection) temperature, reduce the rotational speed of the screw and/or the damming pressure (plasticizing), check the condition of the surface (anti-corrosion layer) of the plasticizing unit and/or the mold, check the mass of a single injection (injection mass (moldings + ingots) should not be less than 10–20% of the mass of the material for the maximum stroke of the injection molding machine, check the alloy path in the cylinder and the mold to see whether there are any residual material spots
**Lines at the Injection Point**		
drawback	cause	corrective action
the parts have fibers at the injection point	temperature in the gate area too high, nozzle temperature too high, inadequate cooling, gate diameter too large, nozzle tip too retracted, inadequate pressure and holding time	try to adjust the nozzle temperatures according to the material, increase die cooling, check the diameter of the gateways, check the nozzle seats, reduce both the back pressure and the screw speed of the injection molding machine, increase hold downtime
**Concentric Tracks Near the Injection Point**		
drawback	cause	corrective action
the profile has matte concentric rings around the injection point	gate too small, injection speed too low, injection or mold temperature too low	increase injection speed, increase mold temperaturę, increase the gate area temperature, increase back pressure
**Traces of Ejectors**		
drawback	cause	corrective action
deformation from the side of pushing	errors in the push system, excessive ejector pressure, wrong push position, insufficient cooling time, excessive injection pressure, mold temperature too high	reduce the pressure on the ejectors, reduce injection pressure, check and, if necessary, change the ejection system, extend the cooling time, lower the mold temperature
**Deformation on Removal**		
drawback	cause	corrective action
delamination on the surface of moldings resulting from insufficient adhesion of the plates of the hardened material	excessive injection speed, the low temperature in the gate area, non-uniform cooling, material contamination, bad mix, the presence of moisture in the material	reduce injection speed, reduce the speed of the auger, raise mold temperaturę, increase the temperature in the gate area, check the granulate for moisture or contamination, check the mixture
**Collapses**		
drawback	cause	corrective action
the depressions are depressions on the surface of the molded part	they mainly appear in areas where more alloy is deposited, typically on the side opposite to the rib underneath. The greater the amount of alloy (thickness of the part) causes a local increase in volumetric shrinkage. This pulls the surface layer inside. If the surface layer does not flow, blisters form in place of the collapsing. Sometimes sags are formed right after the part is removed from the mold, when the hot molded core heats up the already cooled outer layers and causes them to soften	check the correct operation of the check valve at the end of the screw, determine the gate closing time by weighing the element or measuring the internal pressure and extend the clamping time, increase the clamping pressure, lower the mold temperature, lower the injection (stop) temperature, reduce the injection speed, extend the seasoning time after injection, increase the gate cross-section, check if the gate is placed in the thin-walled area of the molded part, increase the pressure in the initial pressure phase, immediately after the seat filling phase, ensure intensive cooling of the molded part after its removal from the mold
**Warping**		
drawback	cause	corrective action
initially, immediately after forming, the shape of the molded part complies with that assumed in the design, and after some time, it twists and turns partially around its axis, the surface is corrugated, certain dimensions are shortened, and the angles between the walls are deformed	the reason for this is different shrinkage tendencies (so-called potential shrinkage) in different parts of the molded part. The differences in the amount of shrinkage depend on the differences in the degree of packing of the material in these parts of the compact and on the differences in the orientation of the macromolecules	ensure even filling of the mold, ensure the best possible packing (compaction) of the alloy in the mold cavity, use the high injection and hold pressure, try to make the packing of the alloy along the flow path homogeneous, increase the number of gates, increase the injection speed, ensure even and symmetrical cooling of the molded part, use a more fluid type of material, use a material with lower compression shrinkage (amorphous and filled plastics have lower shrinkage than semi-crystalline and unfilled ones), take into account the warping of the part in the design of the molding cavity. The socket should be designed so that after warping the molding, the molding will obtain the desired shape, e.g., the lenticular shape of the bottom of the seat, which, after warping the molding, causes its bottom to straighten, reduce differences in wall thickness and places of thickenings (alloy accumulation) of the molded part, insert zones in the molded part, such as concavities or convexities, where possible deformations are not visible or do not interfere, stiffen the zones where the molding has a tendency to warp, avoid sharp edges and corners, change the direction of the reinforcement fibers’ orientation
**Tiger Lines**		
drawback	cause	corrective action
“Tiger lines” are shadows that gradually appear on the surface of the moldings, perpendicular to the direction of flow, resembling tiger hair	they are caused by the pulsating flow of the melt, which occurs especially in the processing of multiphase thermoplastic blends (blends)	increase the temperature of the melt and mold, increase the cross-section of the inlets and the wall thickness of the molded part, use a type of plastic with greater fluidity
**Traces of Cold Plastic**		
drawback	cause	corrective action
due to the too low temperature, portions of the polymer melt solidify in the gating system or in the injection nozzle before filling the mold cavity, and then they are injected during the next injection	this is especially true for thin-film or transparent moldings. If the polymer is not molten, it can block the cross-section available to the flowing material. In extreme cases, it may completely block the flow of plastic to the socket. As the cold material does not form a homogeneous mass with the rest of the alloy, the mechanical properties of the part are also negatively affected	increase the temperature of the injection nozzle, reduce damming (plasticizing) pressure, increase the screw return stroke after plasticizing (decompression stroke) so that the alloy does not leak from the injection molding machine nozzle, increase the nozzle cross-section, use a self-closing nozzle, shorten the contact time of the injection molding machine nozzle with the cold mold
**Streaks (Glass Fibers)**		
drawback	cause	corrective action
the streaks of glass fibers can take the form of rough, mottled, and irregular areas on the surface of the compact, as well as certain surface irregularities which create a flow-line shape in this region of the compact	depending on the angle of incidence of the light, these streaks range in appearance from a cloudy matte to a metallic sheen. Glass fiber streaks tend to increase mainly at openings, thickness variations, curvatures, and flow lines. The formation of these defects is influenced by: injection speed, mold and polymer temperature	increase the injection speed, increase the mold temperature, but not above the maximum value recommended by the plastic manufacturer, increase the injection temperature, improve melt homogeneity by increasing the plasticizing pressure (back pressure) and increasing the rotation speed of the screw during dispensing
**Diesl Effect**		
drawback	cause	corrective action
the local dark discoloration appears in the areas where the streams join	the reason for this disadvantage is that it is difficult to push the air out of the mold cavity and compress it through the face of the flowing melt. If the air cannot leave the seat, it heats up so much during this compression that it “scorches” the surface of the material	check the air ducts for patency, reduce the closing force of the injection molding machine, if the cavity is deaerated through the plane of the mold division, reduce the injection speed, lower the injection temperature, introduce additional venting channels, change the injection point so as to ensure that the air leaves the socket through the existing venting channels
**Record Effect**		
drawback	cause	corrective action
the effect of the gramophone record corresponds to the traces of the melt flow lines perpendicular to the injection direction, which take the form of concentric or parallel grooves on the surface of the compact	injection speed too low, periodic stoppage of the alloy flowing through the undercuts, necks, too early switching from the injection phase to the hold-down phase	set the optimal moment of switching the injection (filling) phase to pressure. The switch point should occur before the slots are completely filled (i.e., about 98% filling), increase the injection speed, increase the temperature of the mold wall, but not above the maximum value recommended by the plastic manufacturer, increase the injection temperature and, if necessary, the hot runner temperature, but not above the maximum value recommended by the plastic manufacturer, check whether it is possible to change the injection point of the material into the molding cavity or change the wall thickness
**Stress Corrosion**		
drawback	cause	corrective action
the external or internal scratches on the molded part are caused by stresses which are less than the destructive stresses	the level of internal stress introduced to the injection molded part is significantly influenced by the processing parameters	increase the mold temperature, but not higher than the recommended maximum mold temperature recommended by the material supplier, equalize the temperature of the cooling systems to obtain the same cooling conditions (temperature, cooling rate) on both sides of the part wall, reduce alloy clusters, reduce the clamping pressure, improve the rigidity of the mold structure
**Scales (Dark, Silver)**		
drawback	cause	corrective action
the silver scales are visible on the surface of the molding as silvery or light to dark brown discoloration	the reason is a serious degradation of the material. The released gaseous substances form bubbles that, during the injection phase, reach the wall of the seat, where they are “smeared” on the surface. Light to dark brown discoloration often indicates severe thermal degradation due to oxidation or decomposition (often occurring after the relatively long machine stops with the heat on). Silver streaks, in turn, are usually the result of excessive friction in a limited area, i.e., in a nozzle with a too small cross-section or too thin billets	check that the machine was not stopped before the defect appeared, check that the temperature of the melt leaving the injection nozzle is within the range recommended for processing this material, check that the temperature at the outlet from the hot runner (if it is present in the mold) is within the range recommended for processing this material, check if the plasticizing system is of the right size (the volume of a single injection into the mold should be within 20–30 to 80% of the maximum stroke volume of the injection molding machine), increase or decrease the rotational speed of the screw, reduce damming pressure (plasticizing), shorten the residence time of the alloy in the hot runner by shortening the injection cycle time, reduce the injection speed, check the geometry of the gates, correct the cross-section of the hot runner and injection nozzle, remove, if possible, all narrow sections and zones of sharp bends
**Microcracks**		
drawback	cause	corrective action
The external or internal scratches on the molded part are caused by stresses which are less than the destructive stresses. Local internal stresses between the areas with poorer packing of macromolecules are responsible for the cracking of the compacts	the formation of cracks or cracks is initiated by external stresses, often accompanied by the action of corrosive agents or fracture promoters (tensile or swelling forces increasing the notch effect). The processing parameters have a significant impact on the level of internal stresses introduced into the injection molding	increase the mold temperature, but not higher than the recommended maximum mold temperature recommended by the material supplier, equalize the temperature of the cooling systems to obtain the same cooling conditions (temperature, cooling rate) on both sides of the part wall, reduce alloy clusters, reduce clamping pressure, improve the rigidity of the mold structure
**Delayering**		
drawback	cause	corrective action
the delamination occurring in the moldings consists in the appearance of visible, not having good adhesion plates of the solidified alloy, or matting in the area of the surface	the reason for this insufficient adhesion between the plastic layers is excessive shear (too high shear stress) of the rather cold alloy caused by intensive cooling in the mold (mold too cold). In the case of semicrystalline materials, this may result in the formation of layers having a different crystal structure. In the case of amorphous materials, it can lead to the separation of the mixture components: polymer-sliding additives, pigments	clean the machine thoroughly when changing the material, check the catalog data of the masterbatch used to determine whether it is suitable for the processed material, check the parameter settings before the recently received correct parts, reduce the injection speed and increase the processing temperature (injection, mold)
**Air Bubbles**		
drawback	cause	corrective action
if the air is trapped in the cavity and surrounded by the polymer melt, this defect can be manifested by streaks on the surface and “burns” due to the Diesel effect	then, air bubbles from below the surface of the part	change the flow profile of the alloy in the socket by adding a barrier in the appropriate places, change the injection point, reduce the decompression stroke and its speed of the screw after plasticizing (dosing), improve the feeding of the screw with plastic (the screw should smoothly take the granules from the injection molding machine’s hopper), check whether there is any leakage between the injection molding machine nozzle and the injection channel (cold or hot inlet channel and gate), reduce the dosing zone
**Sediment on the Surface of Forming Cavity**		
drawback	cause	corrective action
the deposits (blooms) on the surface of the molding cavities are caused by the reaction of products released during polymer processing	decomposition products may include degraded polymers or their degradation products or products derived, for example, from the decomposition of flame retardants. Common causes of this defect are poor cavity venting or an excessively high processing temperature	check that the melt temperature at the outlet from the injection molding machine nozzle or at the outlet from the hot runner is in accordance with the recommended temperature for the processed polymer, reduce the rotational speed of the screw, reduce the injection speed, check that the gating system (sizes and cross-sections of channels and gates) complies with the design recommendations, check the position and effectiveness of the venting channels
**Molding Deformation**		
drawback	cause	corrective action
the compact is deformed due to excessive stresses or their inadequate distribution during ejection	this can cause scratches, cracks, or excessive deformation of the fitting. The greatest deformations are located near the ejectors or at the undercuts that are difficult to eject	change (shorten or extend) the cooling time, improve (reduce) the clamping pressure, lower the temperature of the forming cores, set the optimal moment of switching the injection (filling) phase to pressure. The switch point should occur just before the slots are completely filled (approx. 98% filling), lubricate the forming surfaces with an anti-adhesive agent, increase the mold temperature, improve the venting of the molding cavities by facilitating the exhaust of air through appropriate gaps, parting planes, or the use of special inserts in the space between the core and the molded part
**Uneven Shine**		
drawback	cause	corrective action
The gloss of the molded part depends on how well the seat surface is reproduced on it. In the case of sockets with a matt surface, its good mapping usually results in a shape with a lower gloss because the incident rays are scattered in many directions, i.e., at different angles through many rough planes. On the other hand, if the cavity surface is polished, the part usually has a higher gloss	the basic parameters influencing the removal of this defect are those responsible for the solidification of the outer or top layer and its pressure against the mold wall (mold temperature, injection temperature, injection speed and pressing time)	increase the temperature of the mold wall, but not above the maximum value recommended by the plastic manufacturer, increase the clamping pressure, check the pressure-time setting is correct, set the optimal moment of switching the injection (filling) phase to pressure. The switch point should occur just before the slots are completely filled (i.e., about 98% filling), optimize the injection speed, improve melt homogeneity by increasing the plasticizing pressure (back pressure) and increasing the rotational speed of the screw during dispensing
**Insufficient Infusions**		
drawback	cause	corrective action
the causes of the insufficient infusions may be: - too little amount of plastic in a single injection, - leaky screw tip check valve, - injection pressure too low, - too high melt flow resistance in the mold	these resistances result from the melt viscosity, the length and cross-section of the inflow channels and the thickness of the walls of the part	increase the screw injection stroke, delay the point of switching the injection phase to pressure, increase the injection speed, increase the melt and mold temperature, improve the venting of the seat at the end of its filling, reduce the melt flow resistance through the gating channel, reduce the melt flow resistance through the molding cavity, use a more fluid type of material, check and ensure the patency of the venting channels, change the point of plastic injection into the molding cavity
**Over-Infusion**		
drawback	cause	corrective action
the over-infusion may appear on the surface as protruding lamellar “projections”	they appear when the alloy is pressed between the gaps between the halves of the mold closing surfaces, the cores. This occurs, for example, when too little closing force has been applied	increase the closing force of the mold, reduce the injection speed, reduce the maximum injection pressure, reduce the screw stroke in the plasticizing phase (single injection dose), lower the injection (alloy) temperature and the mold, increase the stiffness of forming plates and seats, improve the fit of both mold halves

**Table 2:** A detailed description of the parameters.

Part Name	Flowmeter
Part volume (total)	1338 cm^3^
Nominal wall thickness	1.7 mm
Color change or long glass fiber	Unknown
Tool description	Unknown
Specified clamp tonnage limit	1000 t
(a) Setting process	
Material	Borealis—HB 601 WG (PP)
Material present in Moldflow Database	Supplemental resin: Borealis—DM55 pharm (PP)
Melt temperature	230 °C
Mold temperature	45 °C
Fill time	2.5 s.
Velocity/Pressure transfer (% volume)	98% of volume filled
The result of the analysis	
Maximum flow rate	334 cm^3^/s
Total system pressure	157.4 MPa
Clamp tonnage calculation	1357.4 t
Projected area	2680 cm^2^
Maximum melt front temperature	230.9 °C
Minimum melt front temperature	112.0 °C
Maximum shear rate	65,611 1/s
(b) Relevant supporting data	
General:	Recommended processing:
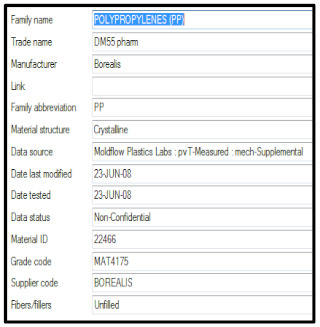	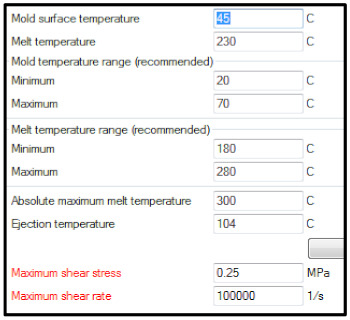

**Table 3:** Image illustrating the flowmeter product inspection plan.

Process Number	Process Name	Machine, Device, Tools for Production	Characteristics			Classification	Methods					Reaction Plan
			Product		Process		Product	Technique	Simple		Method of Control	
			No	Product					Size	Frequency		
									Process Injection process			
—	Visual inspection	Number of form 83,002	1.1	Check material correctness	—	—	System SIGIP	Visual / Operator Team leader!	Once	Each delivery from the warehouse to department	Production control sheet	Isolate materiał (PR2)
—	—	—	1.2	—	Check injection parameters	+	Card of injection parameters	Visual Operator Team leader!	Once	Start shift	Production control sheet	Isolate materiał (PR2)
			1.3	—	Control of correct	+	Card of injection parameters	Process	Once	Everyday	Production control sheet	Isolate material (PR2)
—	—	—	1.4	Check the correctness marking part number and production data on the part	—	+	12,009,975 Self-control instruction CO0035J2D Drawing	Visual Team leader! Operator	1 molded piece	Start production	Production control sheet	Materiał Isolate materiał (PR2)
—	Packing	—	1.9	According to the packing card	—	—	Packaging card	Visual Team leader! Operator	100%	Continuous	—	Isolate materiał (PR2)
—	Visual inspection	—	2.1	Control of all surfaces of the detail	—	+	12,009,975 Self-control instruction C00035J2D Drawing	Visual quality	1 molded piece	3 times on shift	—	Isolate materiał (PR3)
—	Dimension control of part to production approval	—	2.2	Dimension control of part to production approval	—	SPC	C00035J2D Drawing	Bench scales Quality	1 molded piece	3 times on shift	Chart X-R	Isolate materiał (PR3)
—	Dimension control of part to production approval	—	2.3	Dimension control of part to production approval	—	+	21.79 ± 0.1 mm C00035J2D Drawing	Machine 3D Metrology	1 molded piece	Start production every quarter	Dimensional control report	Isolate materiał (PR3)
—	—	—	2.4	Dimension control of part to production approval	—	+	C00035_02D Drawing	Machine 3D Metrology	1 molded piece	Start production every quarter	Dimensional control report	Isolate materiał (PR3)
—	—	—	2.5	Dimension control of part to production approval	—	+	19.8 ± 0.2 mm C00035J2D Drawing	Machine 3D Metrology	1 molded piece	Start production every quarter	Dimensional control report	Isolate materiał (PR3)

**Table 4:** GAA analysis—stocks in closed status.

Product	Data	GAA	Defective	Action	Responsible	Deadline	Status	Done
Corps/Cover GV640 HEX	10	274	joining the material with the loss on the inner wall at the base of the detail (ribbing zone) at the weld line	Make appropriate changes to the injection process	Seters/ Technologist	10	Z	10
Corps/Cover GV640 HEX	10	289	Air bubbles in the front part of the detail as well as in the lower part—the anti-return valve zone	Make appropriate changes to the injection process	Technologist	10		10
Corps/Cover GV640HEX	11	327	Air bubbles on the front end of the body and air bubbles at the weld line and/or the fold of the wall	Process improvement. Updating patterns with defects in the form of air bubbles marked in order to reduce the number of parts rejected by the operator during current production. Delivery of a set of OK/NOK standards also to the production line. Improvement in the field of detail quality	Technologist	11		12
Corps/Cover GV640 HEX	12	327	Air bubbles occurring mainly at the weld line	Inconsistencies from the launch of production—the process is stabilizing	Technologist	12		12

**Table 5:** GAA analysis—actions in open status—no actions were taken.

Product	Data	GAA	Defective	Action	Responsible	Deadline	Status
Corps/Cover GV640HEX	15	411	Air bubbles on the face of the workpiece. No repeatability of non-conformities. A large number of reject parts during production.	Cleaning of mold degassing	Technologist	16	0 0
Corps/Cover GV640HEX	16	411	Air bubbles on the face of the workpiece. No repeatability of non-conformities. A large number of reject parts during production.	Comment: Week 20 still has air bubbles located mainly at the edge of the lid.	Technologist	16	
Corps/Cover GV640HEX	17	452	The material allowance at the injection point of the detail.	Not closing the nozzle.	Technologist	17	
Corps/Cover GV640HEX	21	411	Air bubbles on the face of the workpiece. No repeatability of non-conformities. A large number of reject parts during production.	Cleaning of mold degassing	Technologist	23	

## Data Availability

Data is contained within the article.
